# Supplementary material for: Molecular and Functional Diversity of Distinct Subpopulations of the Stressed Insulin-Secreting Cell's Vesiculome
Source: Front Immunol. 2020 Sep 30;11:1814. doi: 10.3389/fimmu.2020.01814 (PMC7556286; doi:10.3389/fimmu.2020.01814)
Supplement: Supplementary Figure 1 — Original Western blot images of ER stress markers in MIN6 beta cells. After 30 h of culture, 40 μg of cellular protein lysates were blotted and the expression of markers of ER stress (A) p-eIF2α and (B) CHOP was analyzed by western blotting before (C) reprobing of the membranes to β-actin. [file Data_Sheet_1.zip › Supplementary Table 1.docx]

**Suppl. Table 1. EV- associated insulin**

|  | CTL-EV  (pg/E6 cells) | CK-EV  (pg/E6 cells) | UV-EV  (pg/E6 cells) | HX-EV  (pg/E6 cells) |
| --- | --- | --- | --- | --- |
| AB | 1096 (240 – 18100) | 14750 (6190 – 36900)*** | 2850 (159 - 11800) | 2160 (247 - 23400) |
| MV | 348 (104 – 2510) | 1450 (451 - 3850) | 219 (47 - 1620) | 731 (110 - 2340) |
| sEV | 15 (5 - 38) | 51 (13 – 247)* | 11 (2 - 70) | 19 (2 - 50) |

Median (range) quantities of insulin measured in EV from control and treated MIN6 cells. Data from n=7-12 replicates from independent experiments are shown and compared to control conditions using the Kruskal-Wallis test *(*P*<0.05, ****P*<0.001).
