# Supplementary material for: Molecular and Functional Diversity of Distinct Subpopulations of the Stressed Insulin-Secreting Cell's Vesiculome
Source: Front Immunol. 2020 Sep 30;11:1814. doi: 10.3389/fimmu.2020.01814 (PMC7556286; doi:10.3389/fimmu.2020.01814)
Supplement: Supplementary Figure 1 — Original Western blot images of ER stress markers in MIN6 beta cells. After 30 h of culture, 40 μg of cellular protein lysates were blotted and the expression of markers of ER stress (A) p-eIF2α and (B) CHOP was analyzed by western blotting before (C) reprobing of the membranes to β-actin. [file Data_Sheet_1.zip › Supplementary Table 2.docx]

**Suppl. Table 2. EV- associated cytokines**

|  |  | CTL-EV  (fg/E6 cells) | CK-EV  (fg/E6 cells) | Fold increase |
| --- | --- | --- | --- | --- |
| AB | IFNγ | Low | 243.9 (92.1 – 440.6) | *> 34.9* |
|  | IL-23 | 30.5 (17.2 – 50.0) | 21.5 (16.6 – 68.6) | 1.1 |
|  | IL-27 | Low | 3469.7 (2198.0 – 4885.0) | *> 24.2* |
|  | MCP-1 | Low | 2789 (1977.1 – 3039.2) | *> 357.9* |
|  | TNFα | Low | 45.8 (29.4 – 59.1) | *> 12.4* |
| MV | IFNγ | Low | 50.6 (41.2 – 276.9) | *> 14.2* |
|  | IL-23 | 23.6 (17.1 – 84.9) | 18 (16.6 – 138.5) | 1.0 |
|  | MCP-1 | 7.7 (7.3 – 11.3) | 1058.2 (476.5 – 1534.4) | 127.5 |
|  | TNFα | 3.8 (3.6 – 4.8) | 17.2 (11.1 – 19.1) | 4.0 |
| sEV | IFNγ | Low | 14.8 (14.3 – 40.0) | *> 3.1* |
|  | IL-23 | Low | 28.3 (18.0 – 126.1) | *> 3.3* |
|  | MCP-1 | Low | 145.2 (28.7 – 186.6) | *> 16.4* |
|  | TNFα | Low | 10 (7.4 – 24.5) | *> 3.8* |

Median (range) quantities of cytokines measured in EV from CTL and CK-treated MIN6 cells and average increase (mean) in CK-EV compared to NT-EV calculated for n=3-4 replicates from independent experiments. Fold increases in italics are underestimated calculations including samples below detection threshold (set to threshold values).
