# Supplementary figures and images for: Molecular and Functional Diversity of Distinct Subpopulations of the Stressed Insulin-Secreting Cell's Vesiculome
Source: Front Immunol. 2020 Sep 30;11:1814. doi: 10.3389/fimmu.2020.01814 (PMC7556286; doi:10.3389/fimmu.2020.01814)

A

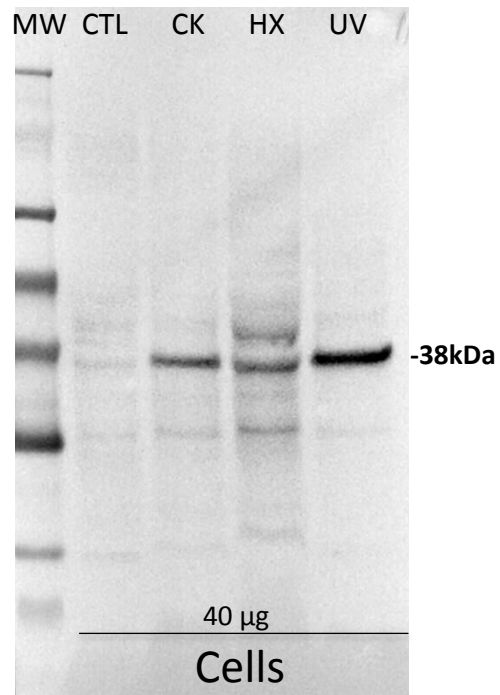

p-eIF2 $\alpha$

B

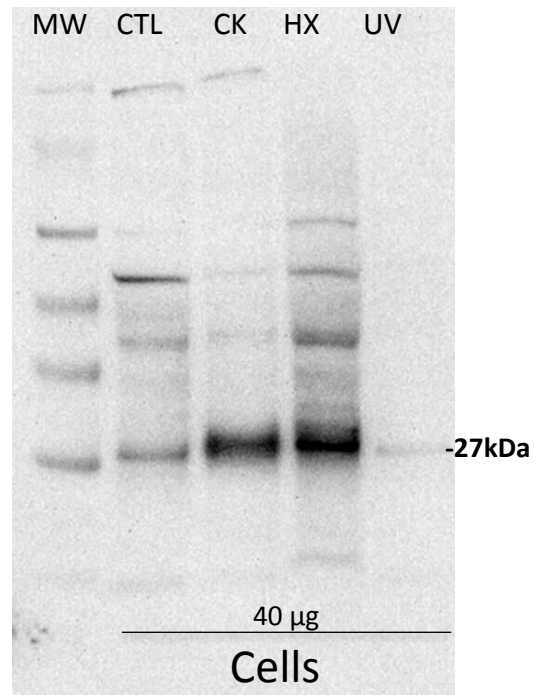

CHOP

C

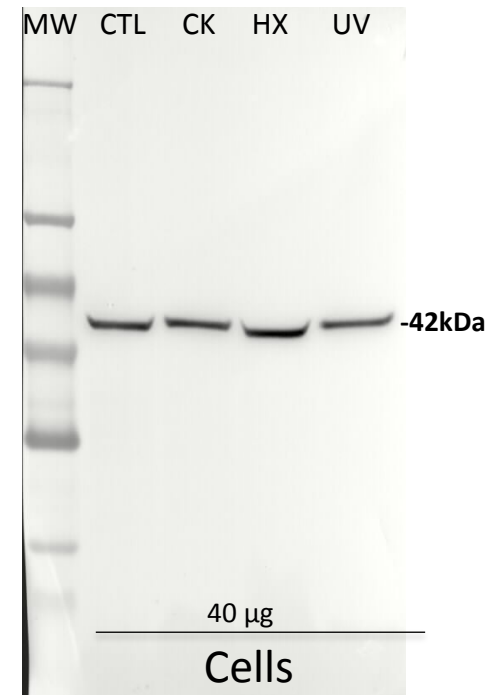

β-actin

Supplement: Supplementary Figure 1 — Original Western blot images of ER stress markers in MIN6 beta cells. After 30 h of culture, 40 μg of cellular protein lysates were blotted and the expression of markers of ER stress (A) p-eIF2α and (B) CHOP was analyzed by western blotting before (C) reprobing of the membranes to β-actin. [file Data_Sheet_1.zip › Supplementary Figure 1.pdf]

A

CD81

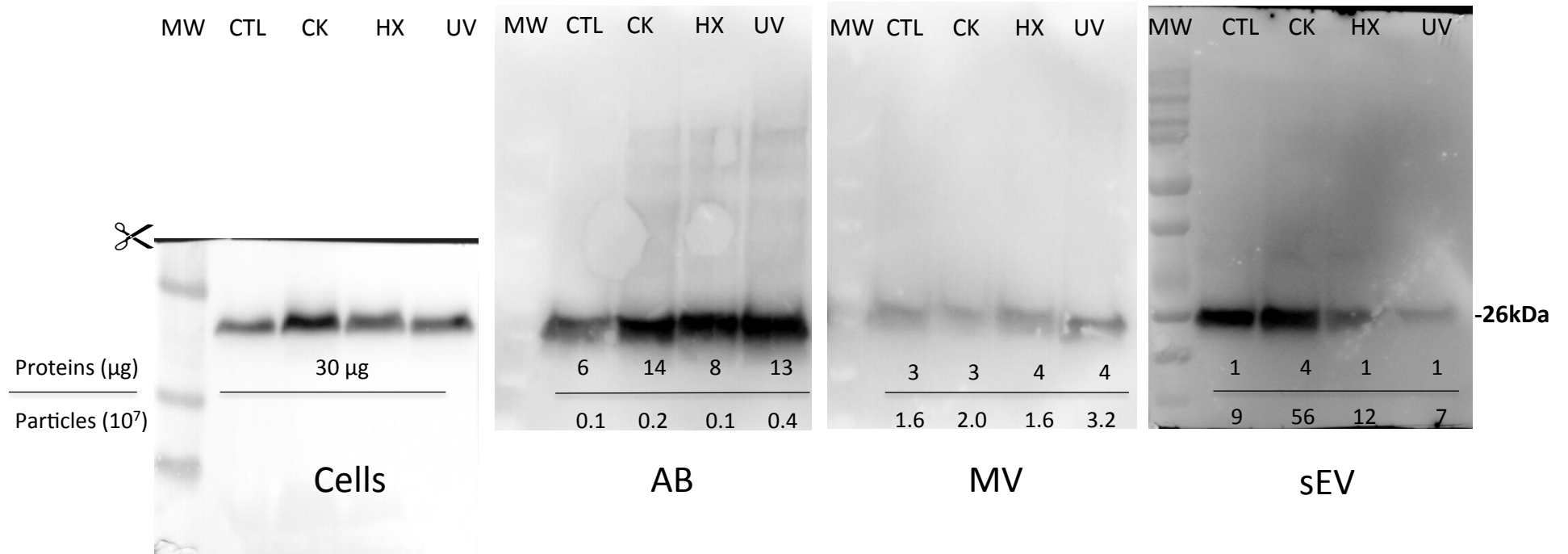

B

# CD63

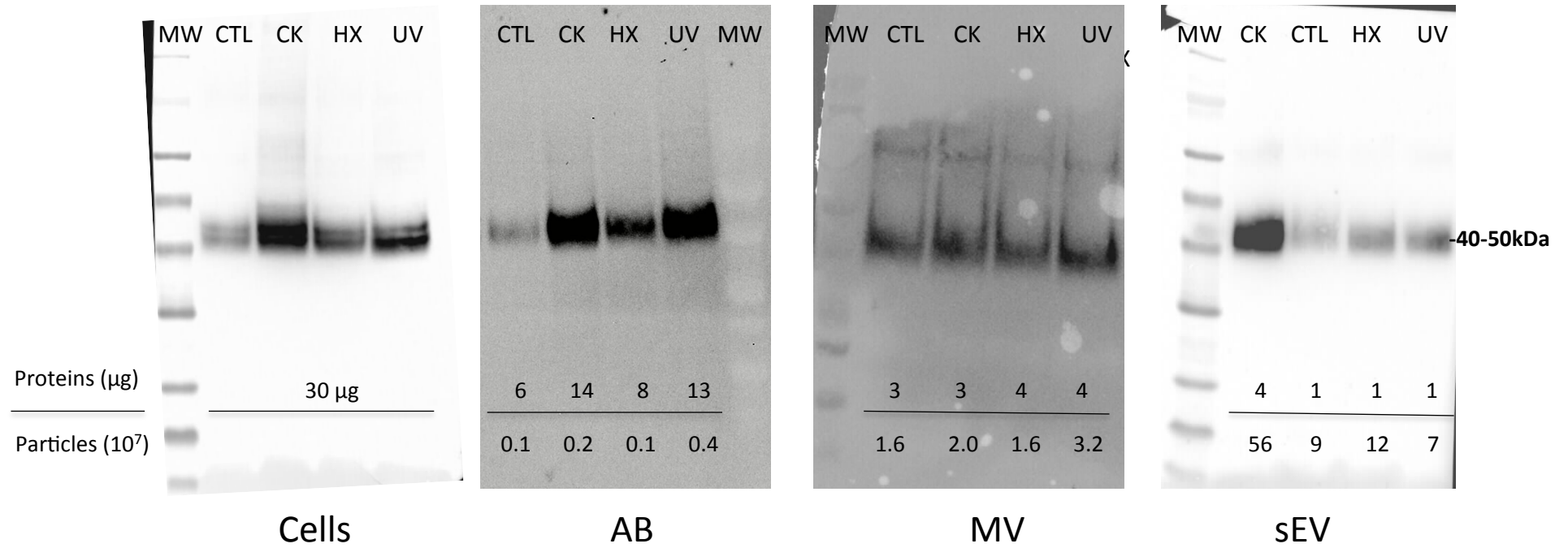

C

CD9

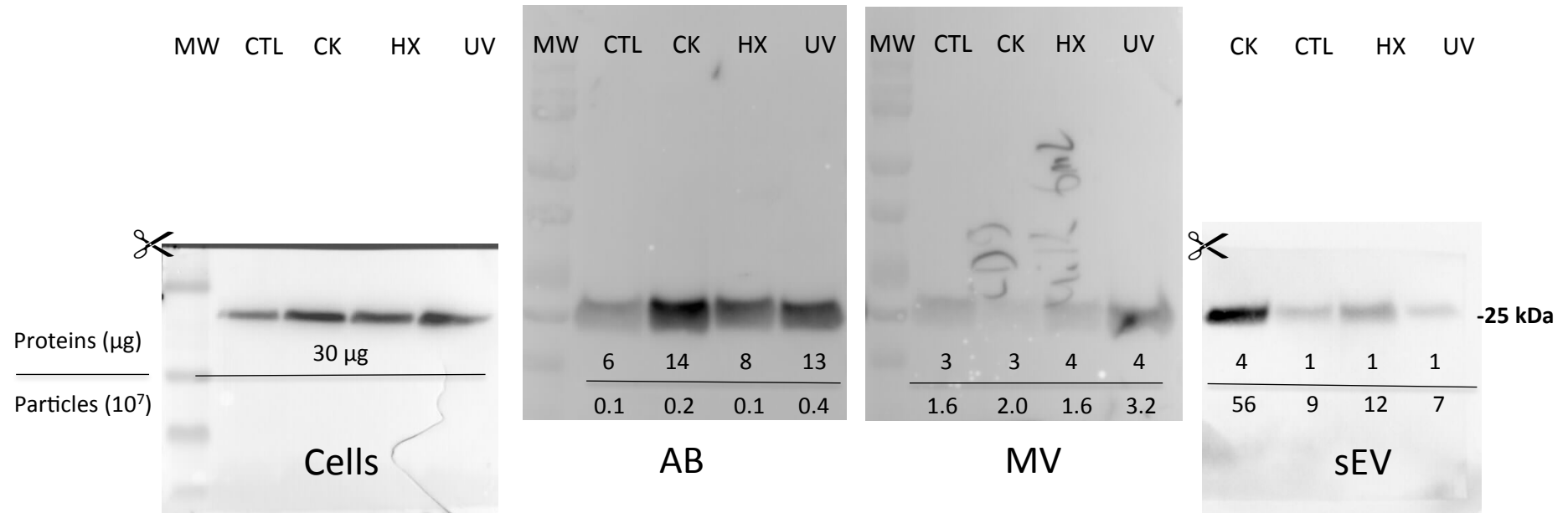

D

## Flotillin-1

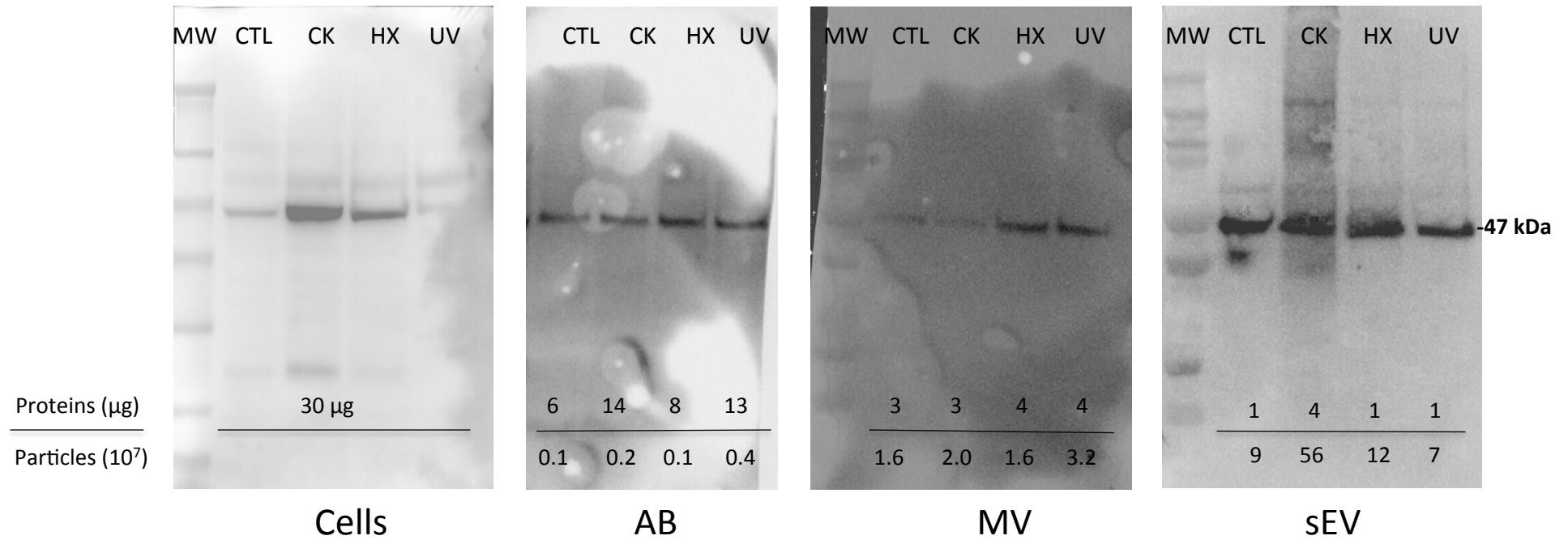

E

$\beta$ -actin

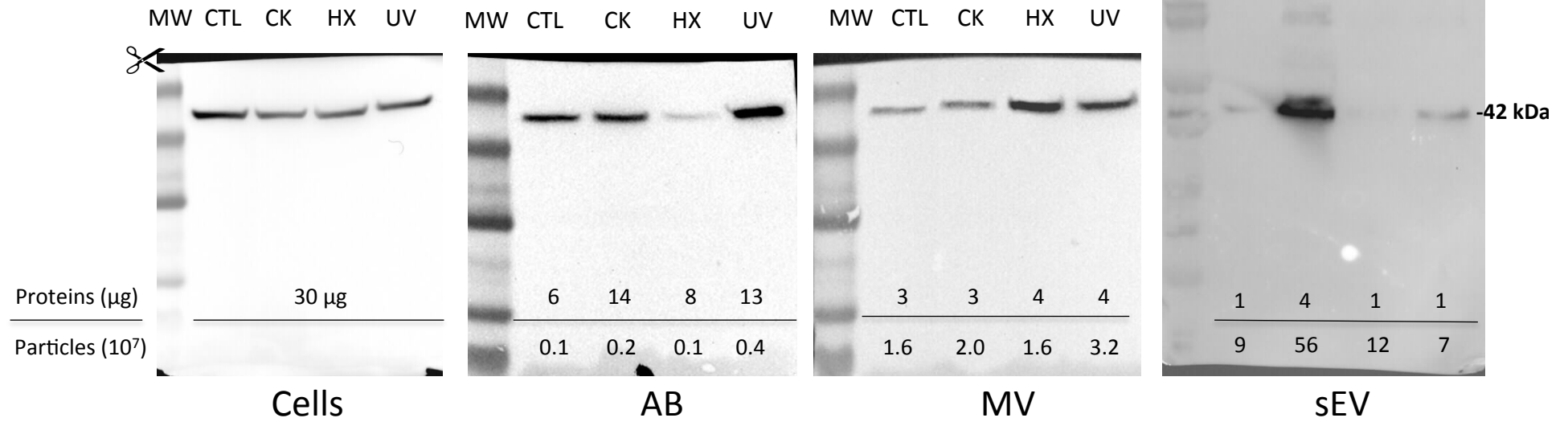

F

## Calnexin

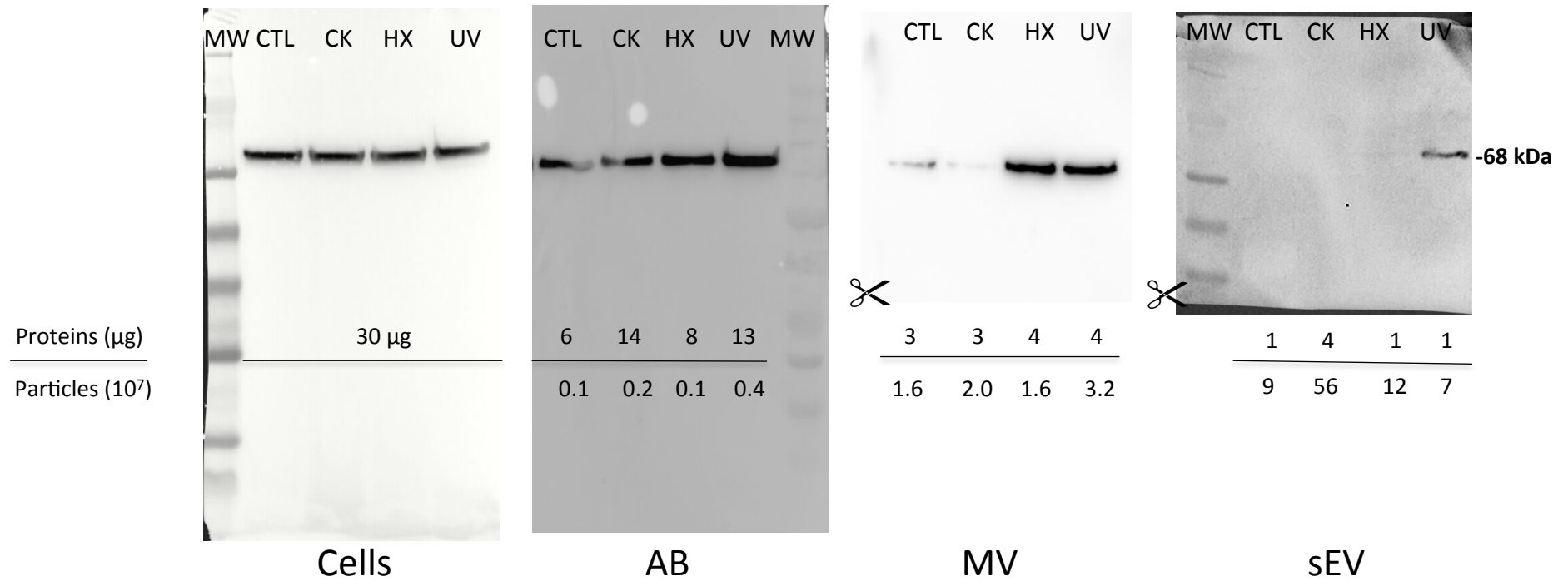

Supplement: Supplementary Figure 1 — Original Western blot images of ER stress markers in MIN6 beta cells. After 30 h of culture, 40 μg of cellular protein lysates were blotted and the expression of markers of ER stress (A) p-eIF2α and (B) CHOP was analyzed by western blotting before (C) reprobing of the membranes to β-actin. [file Data_Sheet_1.zip › Supplementary Figure 3.pdf]

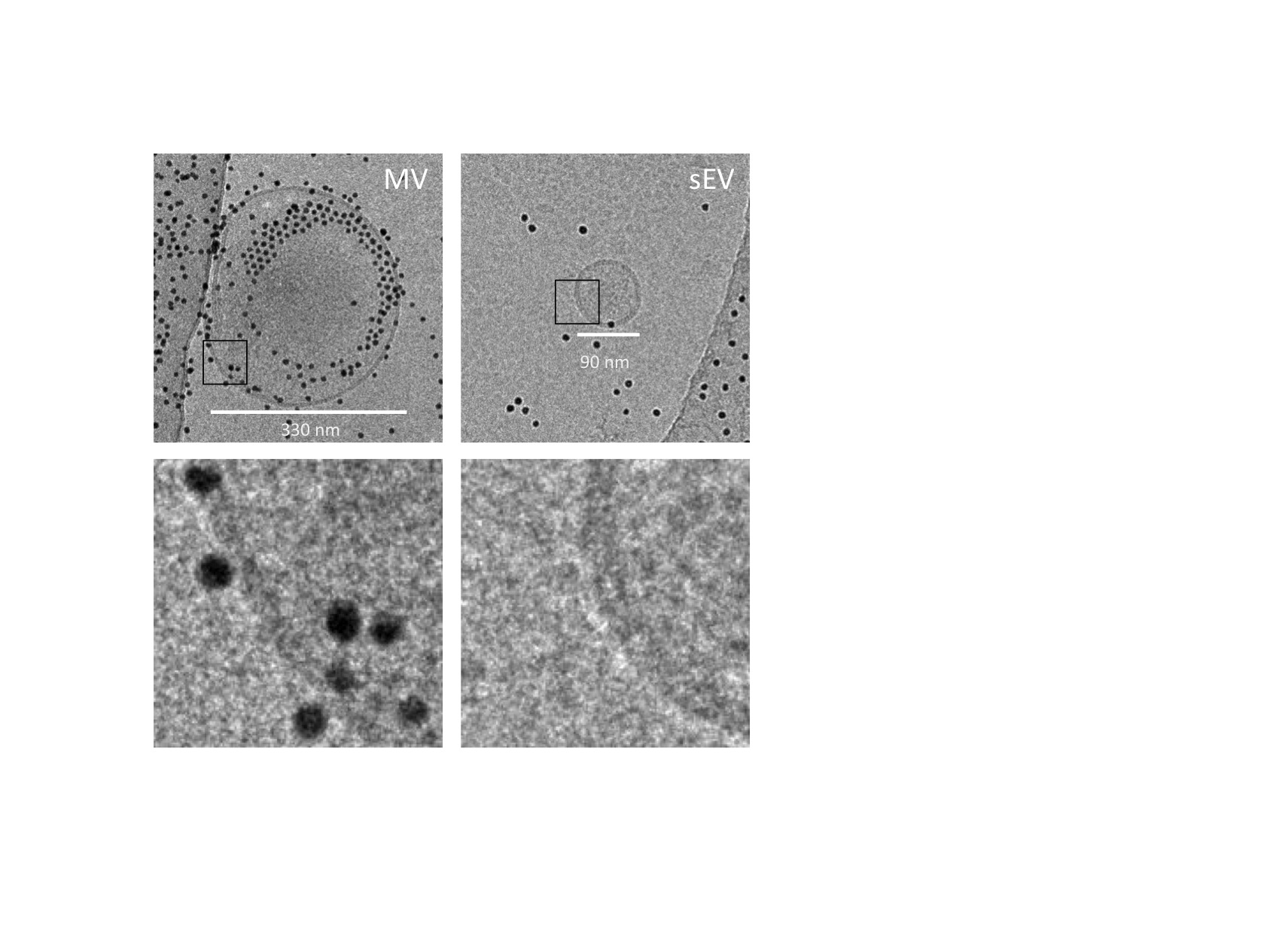

Supplement: Supplementary Figure 1 — Original Western blot images of ER stress markers in MIN6 beta cells. After 30 h of culture, 40 μg of cellular protein lysates were blotted and the expression of markers of ER stress (A) p-eIF2α and (B) CHOP was analyzed by western blotting before (C) reprobing of the membranes to β-actin. [file Data_Sheet_1.zip › Supplementary Figure 4.jpg]

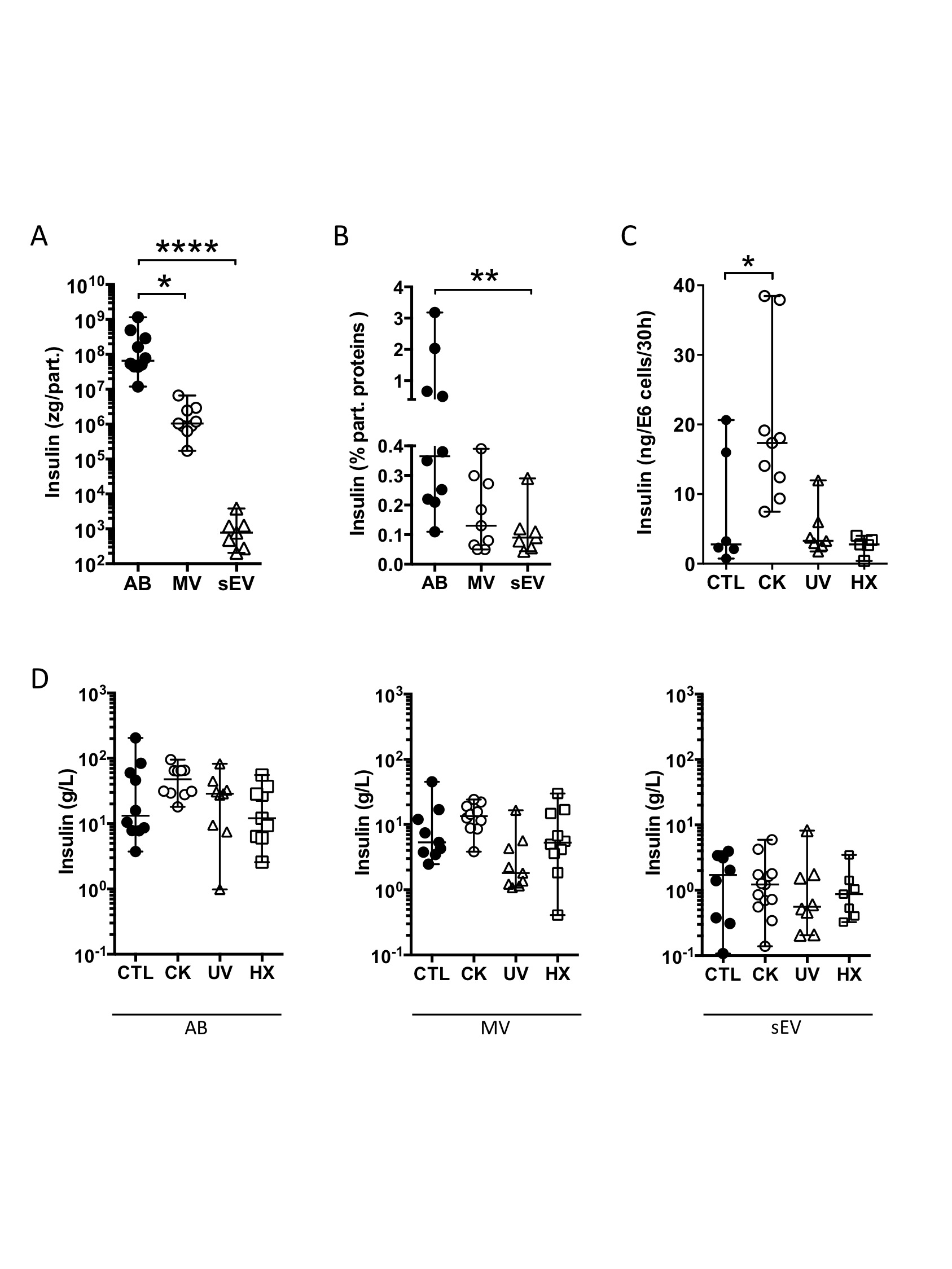

Supplement: Supplementary Figure 1 — Original Western blot images of ER stress markers in MIN6 beta cells. After 30 h of culture, 40 μg of cellular protein lysates were blotted and the expression of markers of ER stress (A) p-eIF2α and (B) CHOP was analyzed by western blotting before (C) reprobing of the membranes to β-actin. [file Data_Sheet_1.zip › Supplementary Figure 5.jpg]

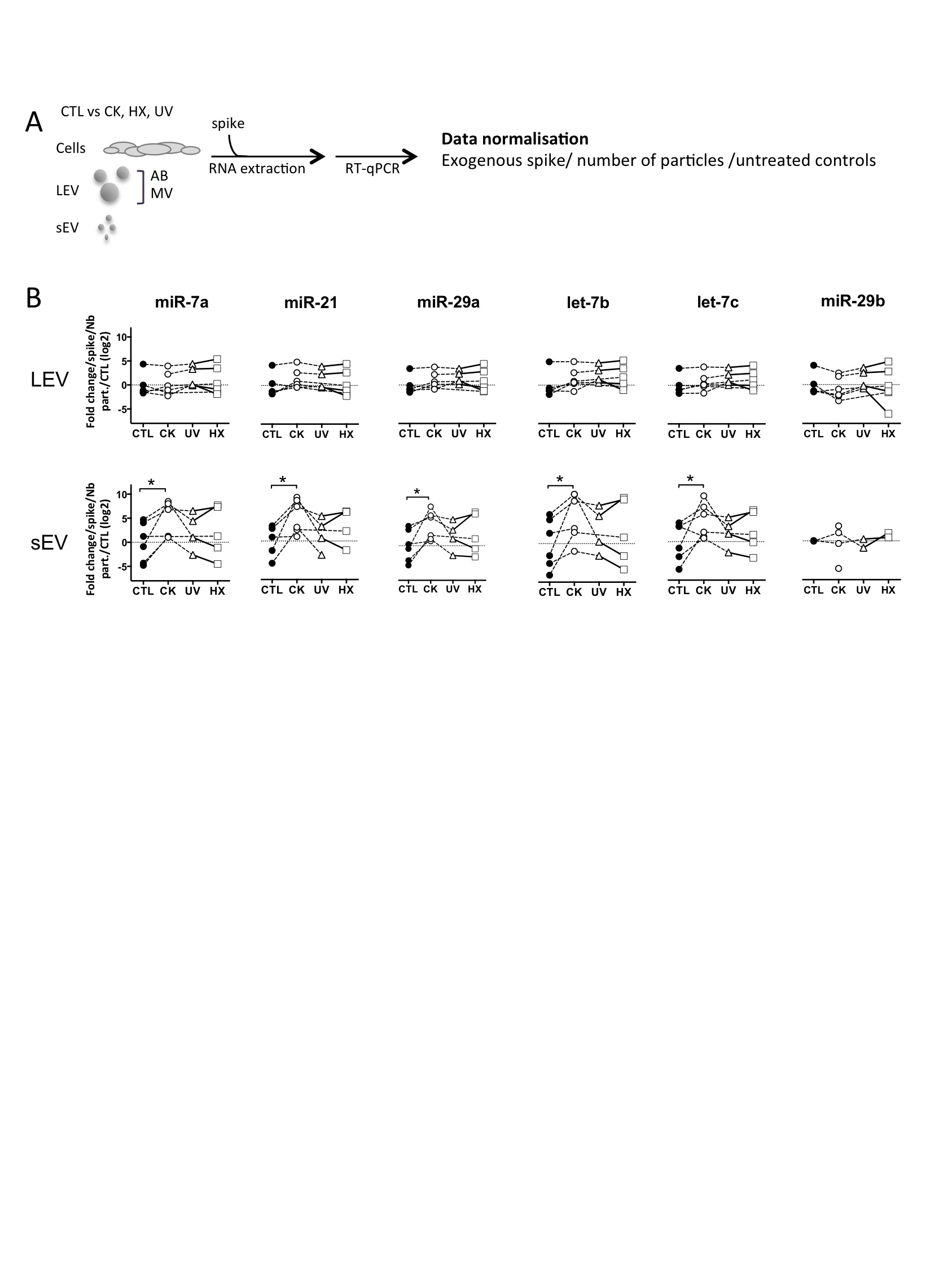

Supplement: Supplementary Figure 1 — Original Western blot images of ER stress markers in MIN6 beta cells. After 30 h of culture, 40 μg of cellular protein lysates were blotted and the expression of markers of ER stress (A) p-eIF2α and (B) CHOP was analyzed by western blotting before (C) reprobing of the membranes to β-actin. [file Data_Sheet_1.zip › Supplementary Figure 6.jpg]

## Slide 1
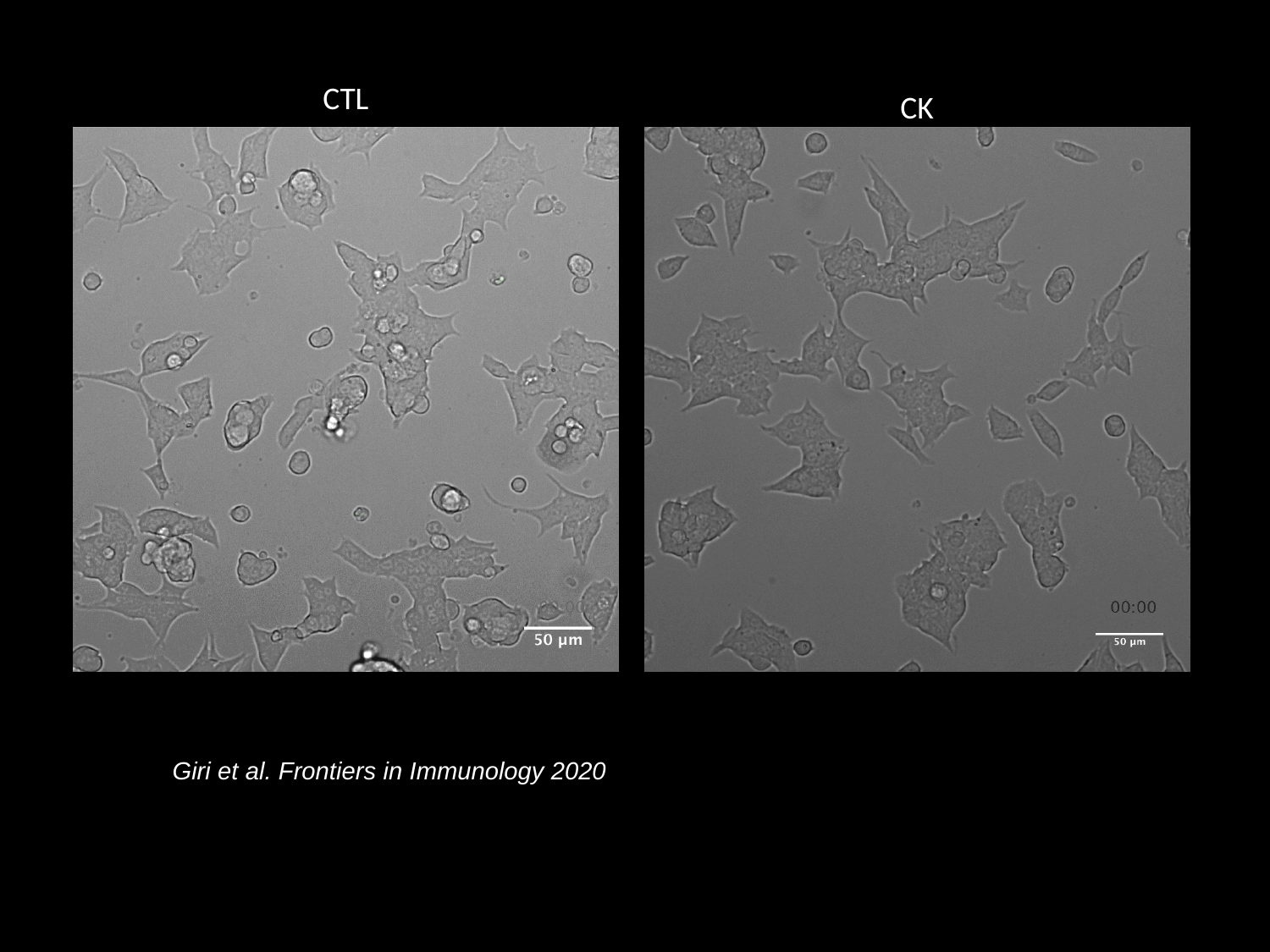

CTL
CK
Giri et al. Frontiers in Immunology 2020

Supplement: Supplementary file 2 [file Presentation_1.pptx]
